# Traumatic Brain Injury, Abuse, and Poor Sustained Attention in Youth and Young Adults Who Previously Experienced Foster Care

**DOI:** 10.1089/neur.2020.0030

**Published:** 2021-02-17

**Authors:** Michael D. Cusimano, Stanley Zhang, Xin Y. Mei, Dana Kennedy, Ashirbani Saha, Melissa Carpino, David Wolfe

**Affiliations:** ^1^ Injury Prevention Research Office, Division of Neurosurgery, Li Ka Shing Knowledge Institute and Trauma and Neurosurgery Program, St. Michael's Hospital, Toronto, Ontario, Canada.; ^2^ Faculty of Medicine and the Dalla Lana School of Public Health, University of Toronto, Toronto, Ontario, Canada.; ^3^ Centre for School Mental Health, Faculty of Education, Western University, London, Ontario, Canada.

**Keywords:** adolescents, adverse childhood events, attention, concussion, traumatic brain injury

## Abstract

Youth and young adults who previously experienced foster care are prone to negative life events, such as physical injuries, and adverse childhood experiences (ACE), such as abuse, neglect, and household dysfunction. The purpose of the present study was to identify the prevalence of traumatic brain injury (TBI), ACE, and poor sustained attention and the associations of these events in this group of vulnerable persons. Participants completed standardized questionnaires on the prevalence of self-reported TBI (TBI) and ACE and performed the Sustained Attention to Response Task (SART) test to measure sustained attention. Chi-squared and Kruskal-Wallis rank-sum tests were used to assess demographic differences and associations between TBI and ACE. Sustained attention was assessed using analysis of variance and linear modeling. Seventy-one participants—46 youth and young adults who previously experienced foster care (vulnerable group) and 25 age-matched healthy controls—completed the standardized questionnaires. Analyses indicated that vulnerable participants reported markedly higher rates of TBI and ACE than healthy controls. Vulnerable persons with TBI reported significantly higher Total ACE scores (*p* = 0.02), were more likely to have a history of family dysfunction (*p* = 0.02), and were more likely to have lived with a mentally ill guardian (*p* = 0.01) than vulnerable persons with no TBI. TBI was significantly associated with Total Errors (*p* = 0.001 and *p* = 0.02) and Omission Errors (*p* < 0.001 and *p* = 0.01) in all participants and in vulnerable participants, respectively, after adjusting for education level.

## Introduction

Traumatic brain injury (TBI) is a major cause of mortality and morbidity in all populations, including children and young adults, worldwide.^1–3^ Post-injury cognitive and psychosocial short-term changes, such as irritability, impulsivity, and aggression, are common and impair recovery.^4,5^ Increasing evidence suggests that the long-term cognitive and behavioral changes resulting from TBI can be associated with violence, adverse social and health behaviors, and further injury. These events may manifest as domestic partner abuse, child abuse, unemployment, criminality, and impaired sustained attention,^6,7^ producing a potential cycle of recurrent injury where the cognitive and social sequelae of TBI pre-dispose victims to future high-risk behaviours and further injury.

This potential cycle of recurrent injury can be observed in foster children as well as those who “age out” of foster care programs at 18 or 19. As of 2011, there were 47,885 and 400,540 children living in foster care in Canada and the United States, respectively.^8,9^ Compared to the general public, youth in foster care are more likely to experience trauma and childhood adversity.^10^ Trauma exposure among youth in foster care is very prevalent (ranging from 80% to 97%), with many youth reporting four or more different types of adverse family experiences, including neighborhood violence, caregiver violence, and living with a mentally ill caregiver or someone with an alcohol or drug problem.^10^ Youth in foster care are also vulnerable to instability and impermanence,^11^ which may, in turn, make them more susceptible to adverse outcomes. In fact, studies have linked placement instability among children in foster care to various adverse outcomes, including behavioral health problems.^12^ One might expect that another adverse outcome would include TBI; however, little is known about the prevalence of TBI and/or its effects in children and young adults who experienced foster care.

Despite limited research exploring the effects of TBI on sustained attention among foster children, results from a study of TBI patients from the general population suggest that TBI may have a negative effect on sustained attention.^13^ According to their results, patients with a closed TBI displayed reduced sustained attention and awareness to errors compared to controls.^13^ Moreover, among these TBI patients, a strong correlation was observed between degree of error awareness and sustained attention capacity, even when controlled for severity of injury.^13^ Despite these findings, further research is needed to better understand the relationship between occurrence of TBI, sustained attention, adverse childhood events like sexual abuse, and foster care.

This study aimed to determine the prevalence of self-reported TBI and adverse childhood experiences (ACE; including physical, sexual, and emotional abuse and neglect) and assess attention task performance in youth and young adults who previously experienced foster care. We also aimed to determine whether associations between these events exist within this population. We hypothesize that persons who previously experienced foster care will have a higher prevalence of TBI and abuse, and reduced sustained attention compared to a control group in the general public, and these events will be significantly associated in this vulnerable population. This work has implications for informing holistic care, support, and child and youth wellness initiatives, as well as injury prevention efforts targeted for this population.

## Methods

### Study participants and ethics approval

A total of 71 persons participated in this study. Forty-six persons who previously experienced foster care were identified and recruited through a child protective services organization (the Catholic Children's Aid Society of Toronto; *N* = 6, 13.1%), and two community youth organizations, including Yonge Street Mission's Evergreen Centre for Street Youth and Covenant House Toronto (*N* = 40; 86.9%). For the purpose of this study, persons who previously experienced foster care were referred to as “vulnerable.”

Twenty-five age-matched controls without a history of foster placement were recruited from St. Michael's Hospital, University of Toronto (Toronto, Ontario, Canada) and local high schools. We referred to these participants as “controls.”

To recruit study participants, we printed advertisements and posted them on bulletin boards at the hospital and various youth organizations, schools, and shelters.

Consent from legal guardians was obtained for all participants under the age of 18. Participants were excluded if they were medically unstable or had comorbidities such as uncontrolled seizures that rendered them unable to complete the tasks required in the study. All participants were compensated in an amount approved by the Research Ethics Board (which was equal to the provincial minimum wage per hour plus travel by public transit).

This work was approved by the Research Ethics Board at St. Michael's Hospital. All participants provided informed consent before being prospectively enrolled in the study.

### Measures

All participants (46 foster participants and 25 controls) completed a General Information Questionnaire, which gathered information on age, sex (male or female), education level, estimated gross income, and self-reported history of TBI. Self-reported TBI status was defined as a “Yes” response to the question: “Have you ever had an injury to the head which knocked you out or at least left you dazed, confused, or disoriented?” For participants who answered “Yes” to this question, the Brain Injury Screening Questionnaire (BISQ) was used to gather *further* details about the reported TBI event.^14^ Participants completed the Adverse Childhood Experiences questionnaire to assess past abuse, neglect, and family dysfunction^15^ and the computer-based Sustained Attention to Response Task (SART) test to assess sustained attention.^16^ Further details about the questionnaires and tests are provided as follows.

### Outcome measures

#### Traumatic brain injury

Self-reported TBI status was assessed using semistructured one-on-one interviews with participants and the BISQ,^14^ a three part 148-item validated instrument which provides information on the number of reported TBIs, their mechanisms, severity (dazed, confused, or loss of consciousness), and associated diagnoses.

#### Adverse childhood experiences

ACE of abuse were self-reported by participants using the Adverse Childhood Experiences questionnaire, which is a 10-question survey divided into three subcategories which represent different forms of childhood adversity: Abuse, Neglect, and Household/Family Dysfunction.^15,17–20^ Participants were asked whether they experienced a specific adverse childhood event described in each question (e.g., verbal abuse, physical abuse, sexual abuse, emotional neglect, etc.). Each “Yes” response is 1 point, for a maximum Total Adverse Childhood Experiences score of 10 points and maximum Adverse Childhood Experiences subcategory score of 3, 2, and 5 for the Abuse, Neglect, and Family Dysfunction subcategories, respectively.^15^

#### Sustained attention

Sustained attention was measured using the SART test—a computerized Go/No-Go task in which a random number between 1 and 9 is presented every 1150 ms.^6,21–24^ The SART has been shown to be a sensitive test to detect attention impairment in patients with mild TBI, regardless of diagnosis.^21^ Each number appears 25 times for a total of 225 stimuli. Participants were instructed to respond to all numbers (go stimuli) by pressing a key except for a single pre-determined no-go stimulus that appears 11% of the time. Dependent variables include Commission Errors (responding to no-go stimuli), Omission Errors (not responding to go stimuli), and Total Errors.^16,25^ We used computerized SART E-prime 2.0 from Psychology Software Tools.

### Statistical analysis

Adverse Childhood Experiences questionnaire results were analyzed for Total Adverse Childhood Experiences scores, subcategory scores, and responses to each individual question. A chi-squared test was used to compare response characteristics of control and vulnerable participants, as well as vulnerable participants with and without TBI. A Kruskal-Wallis rank-sum test was used to assess differences in Total Adverse Childhood Experiences scores by study group followed by a *post hoc* test using the pair-wise Mann-Whitney U test.

SART performance was assessed by the number of Omission, Commission, and Total Errors committed during the trial.^16,26^ Mean Omission, Commission, and Total Errors were compared using an analysis of variance (ANOVA). A *post hoc* test (Tukey's honestly significant difference) was conducted for the paired test after ANOVA. The joint effect of education level and TBI on the attention-related tasks was evaluated using fitted linear models. All statistical analyses were performed using R software (version 3.4.4; R Foundation for Statistical Computing, Vienna, Austria).^27^ A *p* value <0.05 was considered statistically significant.

## Results

### Demographics and traumatic brain injury prevalence

Table 1 summarizes the demographic characteristics of 46 vulnerable participants and 25 controls (*N* = 71). Around half (54%) of the vulnerable participants were male, around one third (30%) had an education level of junior high school or lower, around half (46%) had an income below $10,000, and 61% had self-reported a TBI. The age range for both the vulnerable and control group was 16–28 years. However, most of the vulnerable participants (91%) were between the ages 16 and 26 and all said they were homeless at the time of participation. Of the control group, around one third (32%) were male, more than one third (40%) had an education level of high school or lower, around one fifth (20%) had an income below $10,000, and none self-reported TBI. Most of the control participants (88%) were between the ages 16 and 26. A chi-squared test revealed that control participants had significantly different levels of education (χ^2^ = 21.86, *p* < 0.0001), and they reported significantly different levels of gross income (χ^2^ = 22.65, *p* < 0.0001) than vulnerable participants. Vulnerable participants had significantly higher TBI (χ^2^ = 22.64, *p* < 0.0001) than healthy controls. All other demographic variables did not differ significantly between the two groups.

**Table 1.** Demographic Characteristics of Vulnerable Participants and Healthy Controls (*N* = 71)

Characteristic	Vulnerable**(*N* = 46)*N *(%)	Control**(*N* = 25)*N *(%)
Age categories (years)		
16–18	11 (23.9)	8 (32.0)
19–22	17 (37.0)	3 (12.0)
23–26	14 (30.4)	11 (44.0)
27–30	4 (8.7)	3 (12.0)
Age range (years)	16–28	16–28
Sex		
Male	25 (54.3)	8 (32.0)
Female	21 (45.7)	17 (68.0)
Education level		
Junior high school or less	14 (30.4)	4 (16.0)
High school/GED	28 (60.9)	6 (24.0)
Some college or more	4 (8.7)	15 (60.0)
Gross income		
Decline to answer	11 (23.9)	5 (20.0)
<$10,000	21 (45.7)	5 (20.0)
$10,000–$20,000	12 (26.1)	2 (8.0)
>$20,000	2 (4.3)	13 (52.0)
TBI		
Yes	28 (60.9)	0 (0.0)
No	18 (39.1)	25 (100.0)

GED, General Educational Development; TBI, self-reported traumatic brain injury status.

Among the 28 vulnerable participants who self-reported TBI on the BISQ, 18 (64%) recalled that they had loss of consciousness, 3 (11%) indicated they had been dazed or confused, and the remainder could not recall for certain.

### Abuse prevalence and characteristics assessed using overall Adverse Childhood Experiences questionnaire and its subcategories

Vulnerable participants were further divided by TBI status (no or yes; Table 2). Total Adverse Childhood Experiences scores were not normally distributed. A Kruskal-Wallis test showed that the distribution of Total Adverse Childhood Experiences scores was significantly different among all three study groups (those without TBI, those with TBI, and controls; χ^2^ = 29.12, *p* < 0.001). A *post hoc* test using the pair-wise Mann-Whitney U test showed significant differences between those without and with TBI (*p* = 0.02), those without TBI and controls (*p* = 0.002), and those with TBI and controls (*p* < 0.001). In total, 78% of the vulnerable participants had a Total Adverse Childhood Experiences score >2, as opposed to 9% for controls (Table 2). Among vulnerable participants with TBI, 50% reported Total Adverse Childhood Experiences scores between 8 and 10. None of the vulnerable participants without TBI had a Total Adverse Childhood Experiences score between 8 and 10 (Table 2).

**Table 2.** Distributions of Total ACE Scores and Subcategory Scores in Vulnerable Participants with TBI (No or Yes) and Healthy Controls

	Score	Vulnerable TBI = No**(*N* = 12)*N *(%)	Vulnerable**TBI = Yes**(*N* = 24)*N *(%)	Vulnerable total**(*N* = 36)*N *(%)	Control**(*N* = 22)*N *(%)
Total ACE	0	1 (8.3)	1 (4.2)	2 (5.6)	12 (54.5)
	1	2 (16.7)	1 (4.2)	3 (8.3)	6 (27.3)
	2	1 (8.3)	2 (8.3)	3 (8.3)	2 (9.1)
	3	1 (8.3)	2 (8.3)	3 (8.3)	1 (4.5)
	4	2 (16.7)	1 (4.2)	3 (8.3)	0 (0.0)
	5	1 (8.3)	2 (8.3)	3 (8.3)	0 (0.0)
	6	3 (25.0)	2 (8.3)	5 (13.9)	0 (0.0)
	7	1 (8.3)	1 (4.2)	2 (5.6)	0 (0.0)
	8	0 (0.0)	4 (16.7)	4 (11.1)	1 (4.5)
	9	0 (0.0)	4 (16.7)	4 (11.1)	0 (0.0)
	10	0 (0.0)	4 (16.7)	4 (11.1)	0 (0.0)
Abuse subcategory					
	0	4 (33.3)	4 (16.6)	8 (22.2)	18 (81.8)
	1	1 (8.3)	1 (4.2)	2 (5.6)	1 (4.5)
	2	5 (41.7)	7 (29.2)	12 (33.3)	3 (13.6)
	3	2 (16.7)	12 (50.0)	14 (38.9)	0 (0.0)
Neglect subcategory					
	0	6 (50.0)	6 (25.0)	12 (33.3)	21 (95.5)
	1	4 (33.3)	8 (33.3)	12 (33.3)	1 (4.5)
	2	2 (16.7)	10 (41.7)	12 (33.3)	0 (0.0)
Family dysfunction subcategory					
	0	1 (8.3)	3 (12.5)	4 (11.1)	14 (63.6)
	1	5 (41.7)	3 (12.5)	8 (22.2)	6 (27.3)
	2	3 (25.0)	3 (12.5)	6 (16.7)	1 (4.5)
	3	3 (25.0)	3 (12.5)	6 (16.7)	0 (0.0)
	4	0 (0.0)	5 (20.8)	5 (13.9)	0 (0.0)
	5	0 (0.0)	7 (29.2)	7 (19.4)	1 (4.5)

ACE, Adverse Childhood Experiences questionnaire; TBI, self-reported traumatic brain injury status.

Within vulnerable participants, persons with TBI reported higher Family Dysfunction subcategory Adverse Childhood Experiences scores (χ^2^ = 5.0618, *p* = 0.02) than persons without TBI. Abuse and Neglect subcategory scores did not differ significantly between vulnerable participants with and without TBI (Table 2).

### Associations between specific adverse childhood experiences and traumatic brain injury in vulnerable participants

Table 3 summarizes the responses to individual ACE questions across study groups. Participants who responded partially to the Adverse Childhood Experiences questionnaire were included if they had responded to at least one question. Controls had a significantly lower proportion of “Yes” responses than vulnerable participants on every question (*p* < 0.001), reflecting less exposure to all forms of ACE. Within vulnerable participants, persons who reported TBI had a significantly higher proportion of “Yes” responses to all questions (*p* < 0.001) than persons without TBI. As well, persons with TBI had significantly higher positive response (*p* = 0.01) to living with a mentally ill guardian than persons without TBI. Responses to other ACE questions did not differ significantly between vulnerable participants with and without TBI.

**Table 3.** Responses to Individual ACE Questions in Vulnerable Participants with TBI (No or Yes) and Healthy Controls

		Vulnerable TBI = No	Vulnerable TBI = Yes	Vulnerable total	Control*N *(%)
		N (%)	N (%)	N (%)	
Verbal abuse					
	No	5 (38.5)	7 (25.9)	12 (30.0)	20 (83.3)
	Yes	8 (61.5)	20 (74.1)	28 (70.0)	4 (16.7)
Physical abuse					
	No	6 (46.2)	9 (33.3)	15 (37.5)	21 (91.3)
	Yes	7 (53.8)	18 (66.7)	25 (62.5)	2 (8.7)
Sexual abuse					
	No	11 (84.6)	13 (48.1)	24 (60.0)	23 (95.8)
	Yes	2 (15.4)	14 (51.9)	16 (40.0)	1 (4.2)
Emotional neglect					
	No	6 (46.2)	9 (33.3)	15 (37.5)	23 (95.8)
	Yes	7 (53.8)	18 (66.7)	25 (62.5)	1 (4.2)
Physical neglect					
	No	10 (76.9)	15 (55.6)	25 (62.5)	24 (100.0)
	Yes	3 (23.1)	12 (44.4)	15 (37.5)	0 (0.0)
Divorced/separated parents					
	No	5 (38.5)	9 (33.3)	14 (35.0)	17 (70.8)
	Yes	8 (61.5)	18 (66.7)	26 (65.0)	7 (29.2)
Witnessed domestic abuse					
	No	9 (75.0)	9 (37.5)	18 (50.0)	23 (95.8)
	Yes	3 (25.0)	15 (62.5)	18 (50.0)	1 (4.2)
Living with drug/alcohol abuser					
	No	7 (58.3)	12 (46.2)	19 (50.0)	22 (91.7)
	Yes	5 (41.7)	14 (53.8)	19 (50.0)	2 (8.3)
Living with mentally ill guardian^*^					
	No	10 (83.3)	9 (34.6)	19 (50.0)	20 (87.0)
	Yes	2 (16.7)	17 (65.4)	19 (50.0)	3 (13.0)
Household member went to prison					
	No	9 (75.0)	13 (50.0)	22 (57.9)	22 (95.7)
	Yes	3 (25.0)	13 (50.0)	16 (42.1)	1 (4.3)

The following *p* values are for differences between the vulnerable TBI = No and vulnerable TBI = Yes groups.

^*^ χ^2^ = 5.9679, *p* = 0.01.

ACE, adverse childhood experiences; TBI, self-reported traumatic brain injury status.

### Poorer sustained attention in vulnerable participants with traumatic brain injury

**FIG. 1.**
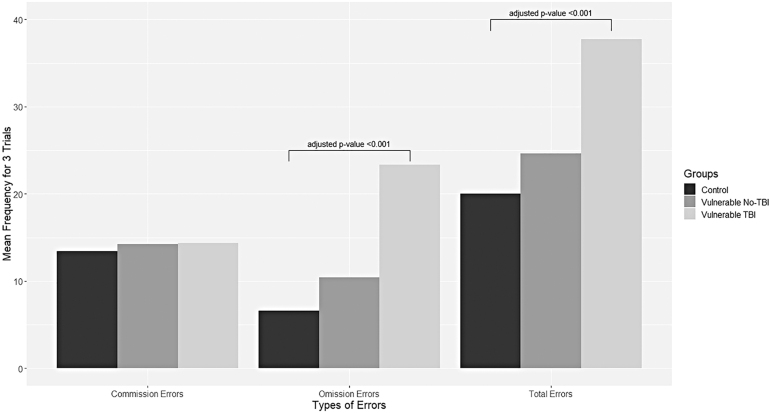
SART performance of vulnerable participants with or without TBI and control participants. SART, Sustained Attention to Response Task; TBI, self-reported traumatic brain injury status.

Figure 1 illustrates SART performance of vulnerable participants and control participants. A *post hoc* test revealed that vulnerable participants with TBI had significantly higher mean Total Errors compared to controls (*p* = 0.001), and mean Omission Errors were also significantly different in vulnerable participants with TBI compared to controls (*p* < 0.001), after adjusting for education level.

### Association between overall and individual adverse childhood experiences questions and sustained attention in vulnerable participants

The joint effect of all components of the Adverse Childhood Experiences questionnaire and TBI on the attention-related tasks was evaluated. An ANOVA test conducted on the fitted linear regression model (residuals for this model were found to be normally distributed) using Omission Errors as the response variable, with TBI and answers to the 10 ACE questions as covariates, found that TBI has a significant effect (*p* = 0.03) on Omission Errors *after* adjusting for all other covariates in vulnerable participants. However, no such effect was noted for any of the covariates for Total Errors and Commission Errors.

Table 4 summarizes associations between SART performance and TBI and between SART and individual ACE questions in vulnerable participants. Vulnerable participants with TBI had significantly higher mean Total Errors (*p* = 0.02) and Omission Errors (*p* = 0.01) than vulnerable participants who did not report experiencing a TBI, after adjusting for education level. Moreover, vulnerable patients without a history of living with a drug/alcohol abuser had significantly higher Commission Errors (*p* = 0.01). SART performance did not differ based on any other ACE question.

**Table 4.** Associations between SART Performance and TBI and between SART and Individual ACE Questions in Vulnerable Participants

Exposure	N	Total Errors	Omission Errors Mean ± SD	Commission Errors**Mean ± SD
		Mean ± SD		
TBI				
No	16	24.6 ± 11.8	10.4 ± 7.4	14.2 ± 5.4
Yes	28	37.8 ± 20.4^*^	23.4 ± 17.9^**^	14.4 ± 5.9
Verbal abuse				
No	11	32.0 ± 18.0	17.5 ± 14.1	14.5 ± 6.7
Yes	27	34.6 ± 20.3	20.5 ± 18.3	14.1 ± 5.0
Physical abuse				
No	14	32.4 ± 16.6	17.8 ± 13.6	14.7 ± 6.0
Yes	24	34.7 ± 21.2	20.7 ± 18.9	14.0 ± 5.3
Sexual abuse				
No	22	30.2 ± 15.8	15.5 ± 11.8	14.6 ± 5.8
Yes	16	38.9 ± 23.1	25.3 ± 21.4	13.6 ± 5.1
Emotional neglect				
No	14	35.1 ± 19.4	21.1 ± 15.7	14.0 ± 6.1
Yes	24	33.1 ± 19.8	18.8 ± 18.0	14.3 ± 5.3
Physical neglect				
No	24	33.2 ± 18.1	18.1 ± 15.2	15.1 ± 5.6
Yes	14	34.9 ± 22.1	22.3 ± 20.1	12.7 ± 5.1
Divorced/separated parents				
No	13	35.7 ± 18.4	20.5 ± 15.0	15.2 ± 6.3
Yes	25	32.9 ± 20.2	19.2 ± 18.3	13.7 ± 5.1
Witnessed domestic abuse				
No	18	30.1 ± 16.5	15.3 ± 14.0	14.9 ± 5.5
Yes	17	38.6 ± 22.9	25.0 ± 20.1	13.6 ± 5.2
Living with drug/alcohol abuser				
No	18	35.2 ± 16.6	18.8 ± 14.8	16.4 ± 4.6^***^
Yes	19	32.5 ± 22.6	20.6 ± 19.7	11.9 ± 5.4
Living with mentally ill guardian				
No	18	32.8 ± 18.1	18.0 ± 15.0	14.9 ± 5.4
Yes	19	34.7 ± 21.5	21.4 ± 19.4	13.3 ± 5.6
Household member went to prison				
No	21	33.0 ± 20.5	18.8 ± 17.2	14.2 ± 6.0
Yes	16	34.8 ± 19.2	21.0 ± 17.8	13.8 ± 4.9

^*^ *F* = 5.536, *p* = 0.02; ^**^F = 7.565, *p* = 0.01; ^***^F = 7.394, *p* = 0.01.

SART, Sustained Attention to Response Task; TBI, self-reported traumatic brain injury status; ACE, adverse childhood experiences; SD, standard deviation.

## Discussion

### High prevalence of traumatic brain injury and abuse in vulnerable participants

Prevalence of TBI in our group of vulnerable persons was 61%—much higher than the average of 25.9% in the general public as reported in a 2011 meta-analysis.^28^ Although recruitment from youth shelters subjects our study group to sampling bias, this difference in TBI prevalence is remarkable. Similarly, results from our Adverse Childhood Experiences questionnaire are in line with previous reports that found that abuse is much more prevalent in foster children. This is not surprising given that foster children have been found to experience more ACE, such as neglect, domestic violence, and abuse, than children in the general public.^29^ Additionally, we found a strong positive relationship between TBI history and adverse childhood experiences in vulnerable participants. Adverse environmental factors, such as unstable family dynamics and transient living situations, may deprive this vulnerable population of a capable and dedicated guardian who can consistently look out for their well-being.^30^ The high-risk environment, coupled with the increased propensity for foster children to engage in risk-taking behavior,^31,32^ can create situations with a high risk for acquiring TBI and/or experiencing abuse or other adverse events.

### Poor sustained attention and traumatic brain injury in vulnerable participants

Vulnerable participants with TBI performed worse on the SART than vulnerable participants without TBI and controls, suggesting that TBI may be linked to differences in sustained attention. This finding is not surprising and is consistent with those found among non-vulnerable populations.^6,13^ Poorer SART performance was only noted in vulnerable participants with TBI, which further supports the link between poorer sustained attention and TBI. Sustained attention is required to survey potential hazards in the environment and is likely required to prevent injury. This is especially crucial for youth and young adults who experienced foster care and homelessness, given that these persons have been documented to participate in more high-risk activities and encounter more hazards in everyday life than the general public.^31,32^ It is tempting to speculate that poorer sustained attention noted in the vulnerable study participants might be a risk factor for future recurrent TBIs, pre-disposing them to a potential cycle of inattention-injury that can further contribute to the poor long-term health outcomes observed in this population. However, given the cross-sectional nature of our data, the temporality of the association found between TBI and poorer sustained attention cannot be determined. As such, these findings may not be uniquely attributable to TBI and require further study in the future.

Previous studies suggest that the SART is sensitive to impulsive responding.^33–35^ In our study, the participants who lived with a drug/alcohol abuser may have experienced an inhibition of such impulsivity, resulting in the lower Commission Errors observed in Table 4.

### Limitations and future directions

Our study has several limitations. First, given the nature of the data, our results represent associations, and thus a cause-and-effect relationship between TBI, ACE, and sustained attention needs further investigation. As such, the reported results and group differences may not be uniquely attributed to TBI. Our results may be prone to sampling bias given the sample size and recruitment approach. Also, given that all vulnerable participants stated that they were homeless at the time of participation, it is possible that some of our sustained attention results could be confounded by the state of homelessness. Therefore, our results may not be generalizable to the entire foster child population, especially to youth and adults who were raised in foster care, but are not homeless at presentation. Our control group systematically differed on several variables from the participants in the vulnerable group, and so we were unable to match controls on educational level. Whereas we acknowledge the potential confounds and weakness of our control group, we would consider the inclusion of a control group a strength, given the ongoing challenge of working in this area.

A feature of our study was that we had a vulnerable group that was not a clinical sample recruited from a hospital or clinic setting. However as a result, our study relied on self-reports of TBI and adverse childhood experiences of abuse, which may be subject to recall bias. Nevertheless, based on participants' self-report, we did know that most TBI reported by participants occurred during childhood. Social desirability bias and misclassification bias are also possible in our measure of ACE given that youth and young adults may have responded “No” to any given question because of the sensitive nature of the information. Finally, our assessment of sustained attention—which is a multi-dimensional construct—relied solely on a single measure (the SART), and we did not have access to clinical diagnostic or other information on diagnoses of attention-deficit hyperactivity disorder and other attention/learning disorders, which have been linked to poor performance on the SART.^36^ As such, data on other significant measures known to influence executive functions were beyond the scope of our study. Although we acknowledge these limitations, our study provides a detailed look into the associations between TBI, ACE, and sustained attention in a disadvantaged population that is largely underserved. Our work has raised important observations, and it should motivate further study and validation in future research.

## Conclusion

Our findings indicate that former foster care is associated with high frequency of TBI, poorer sustained attention, and ACE of abuse and neglect. We posit that persons living in environments at higher risk for injury and abuse may be more likely to sustain a TBI and be pre-disposed to cycles of further trauma facilitated by alterations in cognitive function such as poorer sustained attention. These cyclical events and environments may play a large role in shaping the poor long-term health and life outcomes observed in this disadvantaged population. In fact, literature on the broad sequelae of abuse suggests that the associations that we identified may extend to other adverse outcomes, such as aggression, social withdrawal, and low socioemotional competence, that may further pre-dispose these persons to adverse health and social outcomes.^37,38^ The complicated web of these events and their temporal course should be further elucidated in future research. Ultimately, a better understanding of the temporality and causal pathways of these events will aid in injury and violence prevention efforts and wellness strategies targeted at this vulnerable population.

## The Canadian Brain Injury and Violence Research Team

Michael D. Cusimano, University of Toronto, St. Michael's Hospital; Blaine Hoshizaki, University of Ottawa; Robert Mann, University of Toronto, Centre for Addiction and Mental Health (CAMH); Tom Schweizer, St. Michael's Hospital; David Wolfe, University of Western Ontario, Centre for Addiction and Mental Health (CAMH); Mark Asbridge, Dalhousie University; Shree Bhalerao, St. Michael's Hospital; David Clarke, Dalhousie University Angela Colantonio, University of Toronto; Paul Comper, Toronto Rehabilitation Institute; Wendy Cukier, Ryerson University; Jim Cullen, Centre for Addiction and Mental Health (CAMH) David Delay, University of Manitoba; Peter Donnelly, University of Toronto; Simon Graham, Sunnybrook Hospital, University of Toronto; Jeffrey Hoch, University of Toronto; Stephen Hwang, St. Michael's Hospital; Eric Vaz, Ryerson University; Eric Roy, University of Waterloo Aron Shlonsky, University of Toronto; Charles Tator, Toronto Western Hospital; Lorne Tepperman, University of Toronto; Jane Topolovec-Vranic, St. Michael's Hospital; Donald Voaklander, University of Alberta.

## Abbreviations Used

ACE: adverse childhood experiences
ANOVA: analysis of variance
BISQ: Brain Injury Screening Questionnaire
SART: Sustained Attention to Response Task
TBI: traumatic brain injury

## References

[1] Hill, C.S., McLean, A.L., and Wilson, M.H. (2018). Epidemiology of pediatric traumatic brain injury in a dense urban area served by a helicopter trauma service. Pediatr. Emerg. Care 34, 426–430 29851919 10.1097/PEC.0000000000000845

[2] Hyder, A.A., Wunderlich, C.A., Puvanachandra, P., Gururaj, G., and Kobusingye, O.C. (2007). The impact of traumatic brain injuries: a global perspective. NeuroRehabilitation 22, 341–353 18162698

[3] Thurman, D.J., Alverson, C., Dunn, K.A., Guerrero, J., and Sniezek, J.E. (1999). Traumatic brain injury in the United States: a public health perspective. J. Head Trauma Rehabil. 14, 602–615 10671706 10.1097/00001199-199912000-00009

[4] Kocka, A., and Gagnon, J. (2014). Definition of impulsivity and related terms following traumatic brain injury: a review of the different concepts and measures used to assess impulsivity, disinhibition and other related concepts. Behav. Sci. (Basel) 4, 352–370 25431442 10.3390/bs4040352 PMC4287694

[5] Rao, V., Rosenberg, P., Bertrand, M., Salehinia, S., Spiro, J., Vaishnavi, S., Rastogi, P., Noll, K., Schretlen, D.J., Brandt, J., Cornwell, E., Makley, M., and Miles, Q.S. (2009). Aggression after traumatic brain injury: prevalence and correlates. J. Neuropsychiatry Clin. Neurosci. 21, 420–429 19996251 10.1176/appi.neuropsych.21.4.420 PMC2918269

[6] Dockree, P.M., Kelly, S.P., Roche, R.A., Hogan, M.J., Reilly, R.B., and Robertson, I.H. (2004). Behavioural and physiological impairments of sustained attention after traumatic brain injury. Brain Res. Cogn. Brain Res. 20, 403–414 15268918 10.1016/j.cogbrainres.2004.03.019

[7] Stein, B.D., Zima, B.T., Elliott, M.N., Burnam, M.A., Shahinfar, A., Fox, NA., and Leavitt, L.A. (2001). Violence exposure among school-age children in foster care: relationship to distress symptoms. J. Am. Acad. Child Adolesc. Psychiatry 40, 588–594 11349704 10.1097/00004583-200105000-00019

[8] Adoption and Foster Care Analysis and Reporting System. (2011). AFCARS Report #19. U.S. Department of Health & Human Services: Washington, D.C. www.acf.hhs.gov/programs/cb/resource/afcars-report-19 (Last accessed 129, 2021)

[9] Statistics Canada. (2011). Families and households highlight tables, 2011 census. Date modified: 2020-08-04. www12.statcan.gc.ca/census-recensement/2011/dp-pd/hlt-fst/fam/Pages/highlight.cfm?TabID=1&Lang=E&Asc=1&PRCode=01&OrderBy=999&Sex=1&tableID=304 (Last accessed 129, 2021)

[10] Bramlett, M.D., and Radel, L.F. (2014). Adverse family experiences among children in nonparental care, 2011–2012. Natl. Health Stat. Rep. (74), 1–8 24806543

[11] Hyde, J., and Kammerer, N. (2009). Adolescents' perspectives on placement moves and congregate settings: complex and cumulative instabilities in out-of-home care. Child. Youth Serv. Rev. 31, 265–273

[12] Rubin, D.M., O'Reilly, A.L.R., Luan, X., and Localio, A.R. (2007). The impact of placement stability on behavioral well-being for children in foster care. Pediatrics 119, 336–344 17272624 10.1542/peds.2006-1995 PMC2693406

[13] McAvinue, L., O'Keeffe, F., McMackin, D., and Robertson, I.H. (2005). Impaired sustained attention and error awareness in traumatic brain injury: implications for insight. Neuropsychol. Rehabil. 15, 569–587 16381141 10.1080/09602010443000119

[14] Icahn School of Medicine. (1997). Screening and assessment tools for professionals. icahn.mssm.edu/research/brain-injury/resources/screening (Last accessed 129, 2021)

[15] Felitti, V.J., Anda, R.F., Nordenberg, D., Williamson, D.F., Spitz, A.M., Edwards, V., Koss, M.P., and Marks, J.S. (1998). Relationship of childhood abuse and household dysfunction to many of the leading causes of death in adults. The Adverse Childhood Experiences (ACE) study. Am. J. Prev. Med. 14, 245–258 10.1016/s0749-3797(98)00017-8 9635069

[16] Robertson, I.H., Manly, T., Andrade, J., Baddeley, B.T., and Yiend, J. (1997). ‘Oops!’: performance correlates of everyday attentional failures in traumatic brain injured and normal subjects. Neuropsychologia 35, 747–758 9204482 10.1016/s0028-3932(97)00015-8

[17] Anda, R.F., Felitti, V.J., Bremner, J.D., Walker, J.D., Whitfield, C., Perry, B.D., Dube, S.R., and Giles, W.H. (2006). The enduring effects of abuse and related adverse experiences in childhood. A convergence of evidence from neurobiology and epidemiology. Eur. Arch. Psychiatry Clin. Neurosci. 256, 174–186 16311898 10.1007/s00406-005-0624-4 PMC3232061

[18] Dube, S.R., Anda, R.F., Felitti, V.J., Chapman, D.P., Williamson, D.F., and Giles, W.H. (2001). Childhood abuse, household dysfunction, and the risk of attempted suicide throughout the life span: findings from the adverse childhood experiences study. JAMA 286, 3089–3096 11754674 10.1001/jama.286.24.3089

[19] Dube, S.R., Felitti, V.J., Dong, M., Chapman, D.P., Giles, W.H., and Anda, R.F. (2003). Childhood abuse, neglect, and household dysfunction and the risk of illicit drug use: the adverse childhood experiences study. Pediatrics 111, 564–572 12612237 10.1542/peds.111.3.564

[20] Edwards, V.J., Holden, G.W., Felitti, V.J., an dAnda, R.F. (2003). Relationship between multiple forms of childhood maltreatment and adult mental health in community respondents: results from the adverse childhood experiences study. Am. J. Psychiatry 160, 1453–1460 12900308 10.1176/appi.ajp.160.8.1453

[21] Chan, R.C. (2005). Sustained attention in patients with mild traumatic brain injury. Clin. Rehabil. 19, 188–193 15759534 10.1191/0269215505cr838oa

[22] Farrin, L., Hull, L., Unwin, C., Wykes, T., and David, A. (2003). Effects of depressed mood on objective and subjective measures of attention. J. Neuropsychiatry Clin. Neurosci. 15, 98–104 12556579 10.1176/jnp.15.1.98

[23] Manly, T., Robertson, I.H., Galloway, M., and Hawkins, K. (1999). The absent mind: further investigations of sustained attention to response. Neuropsychologia 37, 661–670 10390027 10.1016/s0028-3932(98)00127-4

[24] Wallace, J.C., and Vodanovich, S.J. (2003). Can accidents and industrial mishaps be predicted? Further investigation into the relationship between cognitive failure and reports of accidents. J. Bus. Psychol. 17, 503–514

[25] Levine, B., Schweizer, T.A., O'Connor, C., Turner, G., Gillingham, S., Stuss, D.T., Manly, T., and Robertson, I.H. (2011). Rehabilitation of executive functioning in patients with frontal lobe brain damage with goal management training. Front. Hum. Neurosci. 5, 9 21369362 10.3389/fnhum.2011.00009 PMC3043269

[26] Whyte, J., Grieb-Neff, P., Gantz, C., and Polansky, M. (2006). Measuring sustained attention after traumatic brain injury: differences in key findings from the sustained attention to response task (SART). Neuropsychologia 44, 2007–2014 16682059 10.1016/j.neuropsychologia.2006.02.012

[27] R Core Team. (2017). R: a language and environment for statistical computing. www.R-project.org/ (Last accessed 129, 2021)

[28] Farrer, T.J., and Hedges, D.W. (2011). Prevalence of traumatic brain injury in incarcerated groups compared to the general population: a meta-analysis. Prog. Neuropsychopharmacol. Biol. Psychiatry 35, 390–394 21238529 10.1016/j.pnpbp.2011.01.007

[29] Bruskas, D. (2008). Children in foster care: a vulnerable population at risk. J. Child Adolesc. Psychiatr. Nurs. 21, 70–77 18429837 10.1111/j.1744-6171.2008.00134.x

[30] Lockwood, K.K., Friedman, S., and Christian, C.W. (2015). Permanency and the foster care system. Curr. Probl. Pediatr. Adolesc. Health Care 45, 306–315 26403649 10.1016/j.cppeds.2015.08.005

[31] Altshuler, S.J., and Poertner, J. (2002). The Child Health and Illness Profile—Adolescent Edition: assessing well-being in group homes or institutions. Child Welfare 81, 495–513 12092670

[32] Gramkowski, B., Kools, S., Paul, S., Boyer, C., Monasterio, E., and Robbins, N. (2009). Health risk behavior of youth in foster care. J. Child Adolesc. Psychiatr. Nurs. 22, 77–85 19490278 10.1111/j.1744-6171.2009.00176.x PMC3436904

[33] Helton, W.S. (2009). Impulsive responding and the sustained attention to response task. J. Clin. Exp. Neuropsychol. 31, 39–47 18608658 10.1080/13803390801978856

[34] Helton, W.S., Kern, R.P., and Walker, D.R. (2009). Conscious thought and the sustained attention to response task. Conscious. Cogn. 18, 600–607 19589699 10.1016/j.concog.2009.06.002

[35] Sakai, H., Uchiyama, Y., Shin, D., Hayashi, M.J., and Sadato, N. (2013). Neural activity changes associated with impulsive responding in the sustained attention to response task. PLoS One 8, e67391 23825657 10.1371/journal.pone.0067391 PMC3692459

[36] Skirrow, C., McLoughlin, G., Banaschewski, T., Brandeis, D., Kuntsi, J., and Asherson, P. (2015). Normalisation of frontal theta activity following methylphenidate treatment in adult attention-deficit/hyperactivity disorder. Eur. Neuropsychopharmacol. 25, 85–94 25435084 10.1016/j.euroneuro.2014.09.015

[37] Shaffer, A., Yates, T.M., and Egeland, B.R. (2009). The relation of emotional maltreatment to early adolescent competence: developmental processes in a prospective study. Child Abuse Negl. 33, 36–44 19167069 10.1016/j.chiabu.2008.12.005

[38] Tyrka, A.R., Wyche, M.C., Kelly, M.M., Price, L.H., and Carpenter, L.L. (2009). Childhood maltreatment and adult personality disorder symptoms: influence of maltreatment type. Psychiatry Res. 165, 281–287 19162332 10.1016/j.psychres.2007.10.017 PMC2671800

